# Novel Apoplastic Antifreeze Proteins of *Deschampsia antarctica* as Enhancer of Common Cell Freezing Media for Cryobanking of Genetic Resources, a Preliminary Study

**DOI:** 10.3390/biom14020174

**Published:** 2024-02-01

**Authors:** Stefania E. Short, Mauricio Zamorano, Cristian Aranzaez-Ríos, Manuel Lee-Estevez, Rommy Díaz, John Quiñones, Patricio Ulloa-Rodríguez, Elías Figueroa Villalobos, León A. Bravo, Steffen P. Graether, Jorge G. Farías

**Affiliations:** 1 Department of Chemical Engineering, Universidad de La Frontera, Av. Francisco Salazar 01145, P.O. Box 54D, Temuco 4811230, Chile; stefania.short@ufrontera.cl (S.E.S.); mauricio.zamorano@ufrontera.cl (M.Z.); c.aranzaez01@ufromail.cl (C.A.-R.); 2 Faculty of Health Sciences, Universidad Autónoma de Chile, Av. Alemania 1090, Temuco 4810101, Chile; manuel.lee@uautonoma.cl; 3 Faculty of Agricultural and Environmental Sciences, Universidad de La Frontera, Av. Francisco Salazar 01145, Temuco 4811230, Chile; rommy.diaz@ufrontera.cl (R.D.); john.quinones@ufrontera.cl (J.Q.); 4 Department of Agronomical Sciences, Universidad Católica del Maule, Av. Carmen 684, Curicó 3341695, Chile; pulloa@ucm.cl; 5 Nucleus of Research in Food Production, Faculty of Natural Resources, Universidad Católica de Temuco, Manuel Montt 056, Temuco 4813302, Chile; efigueroa@uct.cl; 6 Department of Agronomical Sciences and Natural Resources, Universidad de La Frontera, Av. Francisco Salazar 01145, Temuco 4811230, Chile; leon.bravo@ufrontera.cl; 7 Department of Molecular and Cellular Biology, University of Guelph, 50 Stone Rd E, Guelph, ON N1G 2W1, Canada; graether@uoguelph.ca

**Keywords:** antifreeze proteins, apoplastic extract, ice recrystallization inhibition, thermal hysteresis, cryoprotectant

## Abstract

**Highlights:**

Cryopreservation generates ice recrystallization.
*D. antarctica* apoplastic proteins show antifreeze activity.
PMI of *S. salar* sperm can be maintained with AFPs.
High MMP of sperm increases with AFPs.
*D. antarctica* apoplastic proteins act as nonpermeable cryoprotectants.

**Abstract:**

Antifreeze proteins (AFPs) are natural biomolecules found in cold-adapted organisms that lower the freezing point of water, allowing survival in icy conditions. These proteins have the potential to improve cryopreservation techniques by enhancing the quality of genetic material postthaw. *Deschampsia antarctica*, a freezing-tolerant plant, possesses AFPs and is a promising candidate for cryopreservation applications. In this study, we investigated the cryoprotective properties of AFPs from *D. antarctica* extracts on Atlantic salmon spermatozoa. Apoplastic extracts were used to determine ice recrystallization inhibition (IRI), thermal hysteresis (TH) activities and ice crystal morphology. Spermatozoa were cryopreserved using a standard cryoprotectant medium (C+) and three alternative media supplemented with apoplastic extracts. Flow cytometry was employed to measure plasma membrane integrity (PMI) and mitochondrial membrane potential (MMP) postthaw. Results showed that a low concentration of AFPs (0.05 mg/mL) provided significant IRI activity. Apoplastic extracts from *D. antarctica* demonstrated a cryoprotective effect on salmon spermatozoa, with PMI comparable to the standard medium. Moreover, samples treated with apoplastic extracts exhibited a higher percentage of cells with high MMP. These findings represent the first and preliminary report that suggests that AFPs derived from apoplastic extracts of *D. antarctica* have the potential to serve as cryoprotectants and could allow the development of novel freezing media.

## 1. Introduction

Antifreeze proteins (AFPs) are natural biomolecules found in cold-adapted organisms that lower the freezing point of water, allowing the survival of different organisms in freezing conditions. Different proteins with antifreeze activity have been studied as cryoprotectants, which inhibit the growth of ice crystals by altering the structure of ice during freezing [1]; this inhibition has been detected when AFPs have been used for the maintenance of sperm viability after thawing [2]. AFPs have been found in tissues of cold-adapted members of various taxonomic groups, such as fish, insects, bacteria, and plants. In plants, AFPs are found in the apoplastic space, which corresponds to the external space compound of cell walls where water and nutrients are stored [3]. Due to structural complementarity, these proteins have an affinity for ice, thereby inhibiting its growth. Adsorption of AFPs onto ice surfaces has two distinct effects: (i) a noncolligative freezing point depression, which causes a gap between the melting and freezing points of ice; this difference is called thermal hysteresis (TH); and (ii) ice recrystallization inhibition (IRI), where the protein interferes with the migration of ice boundaries, which thermodynamically favors the growth of large ice crystals at the expense of smaller ones [1,4,5,6].

Cryopreservation is a significant method for protecting genetic resources in species with high commercial value, allowing for the conservation of gametes such as spermatozoa from Atlantic salmon (*Salmo salar*) [7]. In the wild, fish such as *S. salar* (as well as amphibians) spawn during autumn and winter, but by manipulating the temperature and photoperiod, spawning can be induced all year round with minimal apparent effects on gamete quality [8]. The same can be said about the seasonal breeding of mammals and birds, which normally would take place only during spring and summer [9]. Cryopreservation can also be applied to accomplish this purpose, allowing good-quality gametes to be collected and stored to be used all year round [10]. The benefits of fish sperm cryopreservation include: (i) storing high-quality sperm for fertilization of oocytes at any time of the year, avoiding the problems of gonadal asynchrony; (ii) establishing databases to carry out a germplasm bank; (iii) optimizing the exchange of semen between reproduction centers; (iv) improving species by allowing the introduction of new genetic lines; and (v) keeping a permanent supply of gametes for optimal use in hatcheries/breeding grounds, or for research [11,12]. However, despite the increasing successes of cryopreservation, this technique may cause serious damage to spermatozoa [13,14]. This is due to the osmotic stress produced by the process, combined with the formation of ice crystals and the toxicity of some cryoprotectants [15,16,17], causing rupturing of the plasma membrane, changes in the mitochondrial membrane potential, and fragmentation of nuclear DNA [18].

The freezing process leads to the formation of large quantities of small ice crystals, some of which will grow/fuse during thawing when samples are warmed at low speed. This highlights the importance of the freezing/thawing rate when preserving different types of cells [19]. In addition, ice recrystallization after thawing has been seen to be detrimental to the integrity of cells, causing damage and contributing to cell death. Thus, establishing adequate amounts of cryoprotectants and optimizing the cooling rates, are essential to avoid the growth of these crystals during the thawing course [20]. In the case of Atlantic salmon spermatozoa, various cryoprotectant compounds have been studied, such as methanol with egg yolk and different combinations of methanol, dimethyl sulphoxide (Me_2_SO), glucose, and sucrose with the addition of bovine serum albumin (BSA) [21], achieving different degrees of improvement. 

Antarctic plants under natural conditions are always in a cold-resistant state because of the climatic conditions of their habitat [22]. *Deschampsia antarctica Desv.* (Poaceae) is a vascular plant species that colonized the maritime Antarctica and exhibits extreme freezing tolerance (LT_50_ −27 °C) [22]. For this reason, it is interesting to try to understand its freezing tolerance and the regulatory mechanism of this process [23]. The presence of ice recrystallization inhibitors in homogenated tissue obtained from plants has been previously reported [24,25]. Highly active AFPs are generally expressed in the leaves of grasses, in the tissue of the roots of herbaceous plants, and in the bark of woody species [24,26]. Antifreeze activity has been previously reported in the apoplastic extracts obtained from leaves of *D. antarctica* [23,27], and it has been demonstrated that cold acclimation induces potent IRI activity, particularly in the apoplast [28]. However, the antifreeze activity has been poorly characterized in this species. Studies have shown that AFPs present in plants have high IRI activity since they display higher effective inhibition of ice recrystallization than AFPs produced by insects [29,30,31]. However, IRI activity is not always proportional to TH activity [2]. Moderately active AFPs bind to the prism and/or pyramidal planes of ice crystals, whereas hyperactive AFPs generally bind to their basal plane [32]. In a comparison of TH and IRI activities between hyperactive insect, bacterial, and fish AFPs with moderately active fish AFPs, Gruneberg et al., (2021) [29] reported that TH hyperactivity of AFPs was not reflected in their IRI activity. These results implied that TH activity does not necessarily correlate with the cryopreservation efficiency of biological samples. Therefore, the utilization of AFPs in cryopreservation cannot be considered based on their TH activity alone [32]. Nevertheless, AFPs may be a useful tool for investigating the role of IRI in cryopreservation and may have potential applications in the improvement of postthawing cell viability [33].

The inhibition of the ice recrystallization process is a key feature that has to be evaluated in order to use plant AFPs as cryoprotectants in the preservation of fish spermatozoa. However, this has not yet been studied. The objective of this work was to characterize the apoplastic extracts of *D. antarctica* by measuring the ice recrystallization inhibition and thermal hysteresis and evaluating its cryoprotective effect on plasma membrane integrity and mitochondrial membrane potential of postthawed Atlantic salmon spermatozoa. 

## 2. Material and Methods

### 2.1. Overview

This study addressed the capability of apoplastic extracts to be used as cryoprotectants of genetic resources. The study was structured to follow three different aspects of the characterization of the AFP-based apoplastic extract from *D. antarctica* that are key for cryopreservation:

(a) Characterization of the antifreeze activity of the apoplastic extract, considering thermal hysteresis activity, ice recrystallization inhibition and ice crystal morphology;
(b) Evaluation of antifreeze activity of the apoplastic extract in presence of a commonly used freezing media for cryopreservation;
(c) Evaluation of cryoprotectant effect of the apoplastic extract in fish spermatozoa.

### 2.2. Extraction of Apoplastic Proteins from D. antarctica

Apoplastic proteins were extracted from the leaves as described by Hon et al., (1994) [34] with modifications according to Short et al., (2020) [25]. Briefly, the leaves of *D. antarctica* were cut into 1.5 cm lengths and infiltrated with a cold solution of 20 mM ascorbic acid and 20 mM CaCl_2_. Once the leaf pieces were vacuum infiltrated, they were placed in a 20 mL syringe barrel, which itself was placed in a 50 mL centrifuge tube, then centrifuged at 4 °C for 30 min at 9000× *g*. The total apoplastic protein content in the extracts was measured as described by Bradford [35]. The calibration curve was performed with BSA as a standard (0.2 mg/mL). Apoplastic extracts were aliquoted and stored at −20 °C until analysis.

### 2.3. Antifreeze Activity Assays

The antifreeze activity was measured in apoplastic extracts of *D. antarctica*. Qualitative confirmation of antifreeze activity was based on changes in ice crystal morphology compared to those crystals grown in a 30% (*w*/*v*) solution of sucrose [34,36]. Measurement of TH activity was carried out according to the method of Patel and Graether [37] with some modifications, using an Otago nanoliter osmometer (Otago Osmometers, Dunedin, New Zealand). A 20 nL drop of apoplastic extract was inserted into a Cargille oil Type B droplet in the cold-stage sample holder. The temperature was lowered to −20 °C until the protein solution froze. Temperature was then adjusted until only a single crystal remained. The photographs of ice crystals were taken using a Canon G10 digital camera. In this method, ice crystals with a hexagonal shape indicated the presence of an ice growth inhibitor, while a circular shape indicates ice crystal growth in a supercooled solution in the absence of an AFP [31]. The IRI measurements were performed as outlined by Hughes et al. (2013) [38] with some modifications. Apoplastic extracts were dissolved in a 30% (*w*/*v*) sucrose solution. The solution (5 μL) was applied between a glass coverslip and the glass slide, placed on a freezing microscope stage, and visualized using a bright-field microscope (model DM RXA 2, Leica Microsystems Wetzlar GmbH, Wetzlar, Germany). Samples were cooled to −40 °C at a rate of 10 °C·min^−1^, after which the temperature was increased to −8 °C at a rate of 30 °C·min^−1^. At this point, the temperature was held constant and photographs were taken to represent 0 min. After 1 h at −8 °C, a second photograph of the samples was taken to represent 60 min. For all images, a 10× objective was used, and the images were digitally captured by a Retiga 1300i camera (QImaging, Surrey, BC, Canada) using the Volocity software package (v.6.2.1; PerkinElmer, Woodbridge, ON, Canada). Additional assays of TH activity were carried on apoplastic extracts, supplemented with a freezing medium consisting of Cortland^®^ (composition per liter is: 1.88 g NaCl, 0.23 g CaCl_2_, 7.2 g KCl, 0.41 g NaH_2_PO_4_, 1 g NaHCO_3_, 0.23 g MgSO_4_·7H_2_O, 1.0 g glucose, 10% (0.02 M) glycol and 10% Tris (0.02 M), pH 9.0; 261 mOsm) [39] diluent and cryoprotectants: dimethyl sulfoxide (Me_2_SO) 1.3 M; glucose 0.3 M and bovine serum albumin (BSA) at 2% (*w*/*v*).

### 2.4. Broodstock

Semen samples were obtained from 12 male breeders of Atlantic salmon (*Salmo salar*) belonging to Hendrix Genetics Aquaculture S.A., a fish farm located at Camino Rinconada Km 6—Sector Catripulli, Curarrehue, Región de La Araucanía, Chile (39°23′17″ S, 71°40′40″ W). The *S. salar* were 3 years old (sexually mature) and had an average weight of 7.5 ± 0.3 kg and a length of 87 ± 0.2 cm. They were kept in 3000 L fiberglass tanks with recycled fresh water (500 L/h) at 10 °C with a natural photoperiod.

### 2.5. Semen Collection and Analysis

Semen was collected using the procedure described by Díaz et al. (2019) [40]. Briefly, males were anesthetized in a 50 L tank with 125 mg/L of tricaine methanesulfonate for 10 min. The urogenital pore was dried, and semen samples were collected with a syringe by abdominal massage and transferred directly to a sterile plastic container at 4 °C. All samples were mixed, obtaining a singular pool with all the gamete samples prior to freezing. Samples contaminated with blood, urine, feces or water were discarded. Immediately after collection, sperm motility and concentration were determined. Motility was assessed by subjective microscopic examination using a phase contrast microscope (Carl Zeiss, Jena, Germany) at 400× magnification; 2 µL of semen diluted to 10 × 10^6^ spermatozoa/mL were placed on a glass slide, and 10 µL of the activator Powermilt^®^ (Universidad Católica de Temuco, Temuco, Chile) at 10 °C was immediately added. Motility was evaluated using values from 0 to 100%, according to Magnotti et al. (2022) [41]. Sperm concentration was determined with a Neubauer hemocytometer in a standard culture medium (Cortland^®^) for fish spermatozoa [42]. Samples that showed >80% motility and sperm concentrations 10 × 10^9^ ± 1.4 spermatozoa/mL were included in this study [42]. 

### 2.6. Cryopreservation Process

Semen was frozen by a modified protocol based on Lahnsteiner et al. (2011) [43]. Frozen semen was diluted at 4 °C in Cortland^®^ medium as described by Figueroa et al. (2013) [44], supplemented with 1.3 M Me_2_SO, 0.3 M of glucose and 2% (*w*/*v*) BSA to establish the standard freezing medium as a positive control. Table 1 shows the different treatments that were made with 0.05 mg/mL of apoplastic extract of *D. antarctica*, which itself was supplemented with permeating cryoprotectants (1.3 M Me_2_SO and 0.3 M glucose), or a nonpermeating cryoprotectant (2% BSA (*w*/*v*)). The dilution ratio was 1:3 (semen: cryoprotectant medium). Aliquots of 0.5 mL were put into plastic straws, and frozen in liquid nitrogen vapor, 2 cm above the liquid nitrogen level, for 10 min before being plunged into liquid nitrogen (−196 °C). Samples were stored in cryogenic tanks for two weeks before thawing for all assays, except for motility assessment, which was measured after 30 days of storage. Straws were thawed in a thermo-regulated bath at 37 °C for 7 s before analysis.

### 2.7. Frozen–Thawed Semen Analysis 

Frozen–thawed semen was evaluated by flow cytometry. The fluorescence analysis was performed on a FACSCanto^TM^ II flow cytometer (BD Biosciences, San Jose, CA, USA). Samples were acquired and analyzed with the FACSDiva^TM^ software (v.6.1.3; Becton, Dickinson and Company, BD Biosciences, San Jose, CA, USA).

The viability of the spermatozoa and the integrity of the cytoplasm membrane were assessed using the LIVE/DEAD Sperm Viability Kit (Invitrogen Inc. Eugene, OR, USA; SYBR- 14/PI dye) as described by Figueroa et al. (2013) [44]. For this, 4 × 10^6^ spermatozoa/mL were resuspended in 250 μL of Cortland^®^ medium. Subsequently, 1 μL of 100 nM SYBR-14 was added and incubated at 10 °C for 10 min. Samples were then incubated with 1 μL of 9.6 µM PI for 10 min at 10 °C. The percentage of cells with membrane integrity was analyzed in each trial with three replicates. 

Changes in the mitochondrial membrane potential (ΔΨm) were determined by using the fluorescent probe JC-1 (5,5′,6,6′-tetrachloro-1,1′,3,3′-tetraethylbenzimidazolylcarbocyanine iodide). This test was performed as per manufacturer instruction for the Mitochondrial Permeability Detection Kit AK-116 (MıT-E-Ψ, BIOMOL International LP, Plymouth Meeting, PA, USA). Briefly, 1 μL of 0.4 μM JC-1 was added to 4 × 10^6^ spermatozoa/mL resuspended in 250 μL of Cortland^®^ medium and then incubated for 15 min at 10 °C in the dark [44]. Samples were subsequently incubated with 1 μL of 9.6 μM PI for 10 min at 10 °C. The analysis in each trial was performed with three replicates.

### 2.8. Postthaw Motility Assessment 

Sperm motility was assessed using a computer-assisted sperm analysis (CASA) system with the ISAS^®^ software (Integrated Sperm Analysis System v.1.0; Proiser, Valencia, Spain). Samples were diluted to a concentration of 10 × 10^6^ spermatozoa/mL. Next, 2 μL droplets of diluted semen were placed on glass slides, and immediately 10 μL of the Powermilt^®^ (Universidad Católica de Temuco, Temuco, Chile) activator at 10 °C was added. Examination was performed in a phase-contrast microscope (Nikon, Eclipse 80i, Tokyo, Japan) at 100× magnification. In each sample, motility was evaluated in triplicates using the adjustments for assessing fish spermatozoa. The parameter settings were 50 frames/s and average path velocity (VAP) > 10 μm/s to classify a spermatozoon as motile. Total sperm motility (MS, %) was recorded.

### 2.9. Statistical Analysis 

All assays were performed in triplicates. Data were expressed as mean ± standard deviation and analyzed with statistical software GraphPad Prism^®^ (v.5.0; GraphPad Software, Boston, MA, USA). One-way ANOVA with Tukey post hoc tests were applied for multiple comparisons, including the thermal hysteresis activity of apoplastic extracts, the plasma membrane integrity and mitochondrial membrane potential analysis. The level of significance was set at *p* < 0.05.

## 3. Results

### 3.1. Antifreeze Activity in D. antarctica Apoplastic Extract

**Figure 1:**
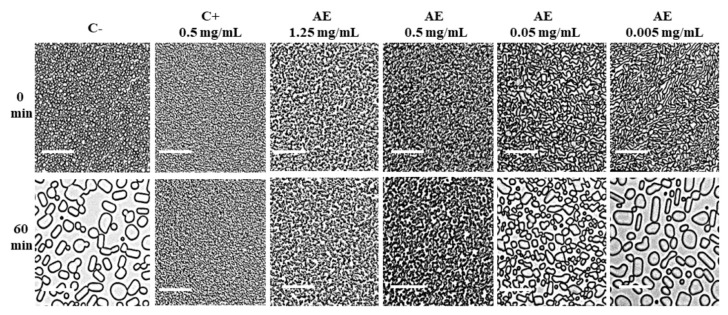
Ice recrystallization inhibition (IRI) activity of controls and apoplastic extracts of *D. antarctica.* C−: Negative control (30% Sucrose); C+: Positive control (type I AFP); AE: Apoplastic extracts. The extracts were serially diluted with the protein concentration shown above each panel. The scale bar represents 25 μm.

**Figure 2:**
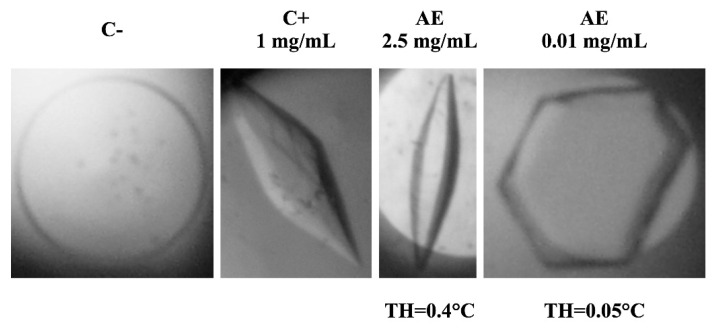
Effect of apoplastic protein concentration on the ice crystal morphology and thermal hysteresis (TH) activity. C−: Negative control (30% Sucrose); C+: Positive control (type I AFP); AE: Apoplastic extracts at two different concentrations.

IRI assays were performed on a stepwise series of dilutions of apoplastic extracts of *D. antarctica*. This assay detects IRI by qualitatively comparing the increase in ice crystal size at 0 and 60 min of incubation of samples at −8 °C (Figure 1). The negative control (samples in the absence of apoplastic proteins) displayed that ice crystals had grown visibly larger after 60 min. A similar growth was observed in samples treated with the most diluted apoplastic extract (0.005 mg/mL). In contrast, the positive control (fish type I AFP) and the concentrated apoplastic extract (0.5 mg/mL) exhibited no visible crystal growth after 60 min, while the 0.05 mg/mL sample showed limited but appreciable inhibition. The experiment revealed that the minimum concentration of apoplastic proteins for detecting IRI activity was 0.05 mg/mL (Figure 1). Following, ice crystal morphology and TH activity were examined in the presence and absence of apoplastic extracts from *D. antarctica* (Figure 2). The ice crystal morphology showed a bipyramidal shape at a higher apoplastic protein concentration, while the shape of the ice crystal exhibited hexagonal morphology at a lower concentration. The TH activity was 0.4 °C and 0.05 °C to 2.5 and 0.01 mg/mL of apoplastic extract, respectively. 

### 3.2. Effect of Freezing Medium on Antifreeze Activity in Apoplastic Extracts of D. antarctica

**Figure 3:**
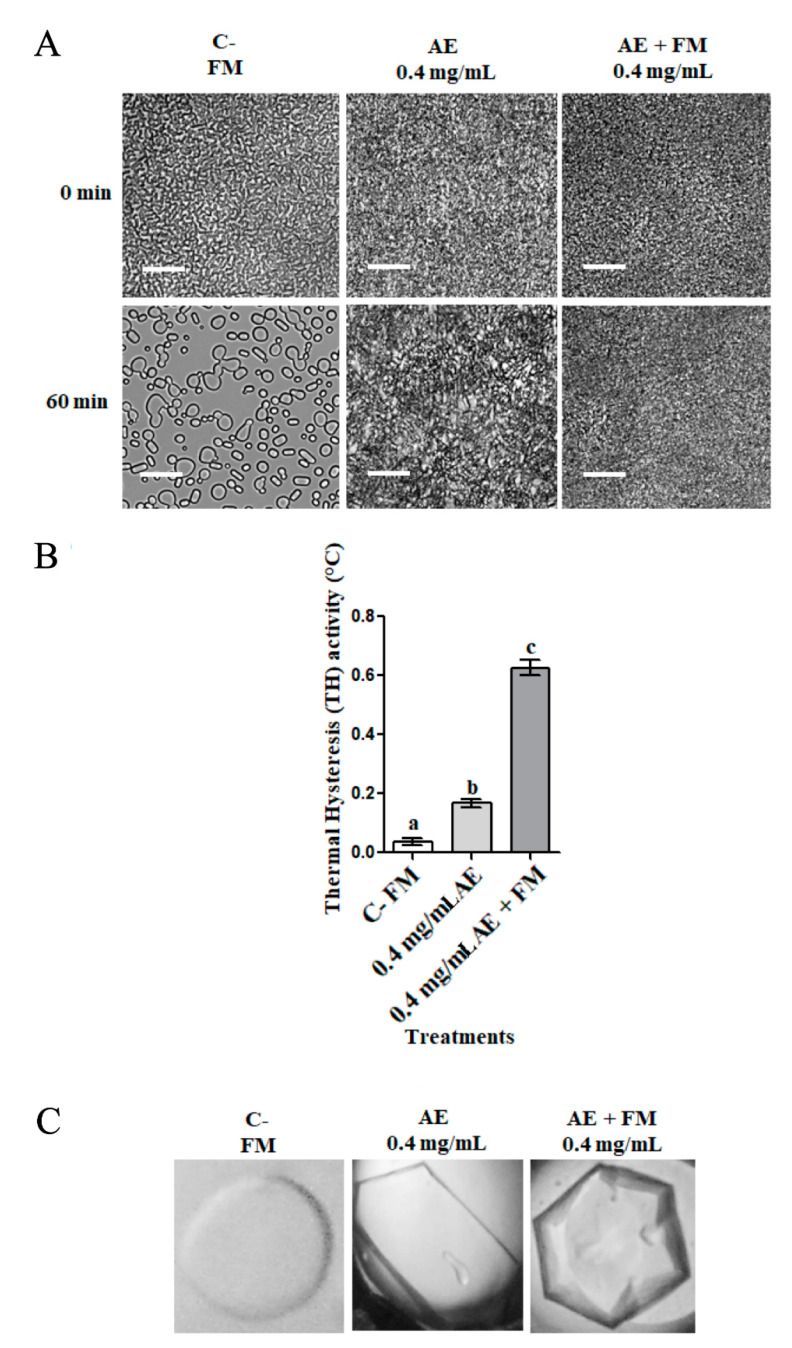
Effect of freezing medium on the antifreeze activity of apoplastic extracts of *D. antarctica*. (**A**) Ice recrystallization inhibition (IRI) activity. (**B**) Thermal hysteresis (TH) activity. (**C**) Ice crystal morphology in presence of apoplastic proteins. C− (FM): Freezing medium with Me_2_SO, glucose and BSA in Cortland^®^ diluent; AE: apoplast extract obtained from plants cold acclimated for two weeks; AE + FM: apoplast extract obtained from plants cold acclimated for two weeks supplemented with freezing medium. One-way ANOVA and Tukey’s post hoc tests were applied for multiple comparisons. Different letters indicate significant differences, *p* < 0.05, *n* = 3.

In order to identify whether the freezing medium decreases or increases the antifreeze activity of apoplastic proteins of *D. antarctica*, and hence determine the ability of apoplastic proteins to act as cryoprotectants, the antifreeze activity was measured in extracts supplemented with freezing medium used for cryopreservation of *S. salar* spermatozoa (Figure 3). The apoplastic extracts supplemented with freezing medium (AE + FM) showed a high capacity to inhibit the growth of ice crystals, similar to extracts without cryopreservation medium (AE); while the extract-free freezing medium (C−) showed no signs of inhibition of the recrystallization of ice (Figure 3A). Regarding TH activity, significant differences were observed between the different conditions; AE + FM improved their TH activity compared to AE, increasing TH from 0.2 °C to 0.63 °C (Figure 3B). Finally, the morphology observed in the ice crystals in the presence of AE + FM showed a slight difference with respect to the ice crystal in the presence of AE, specifically in Figure 3C, right panel, we observe a hexagonal bipyramid shape, which is characteristic of the presence of an antifreeze protein that interacts with both the prism faces and pyramidal planes.

### 3.3. Cryoprotective Effect of Apoplastic Extracts of D. antarctica 

**Figure 4:**
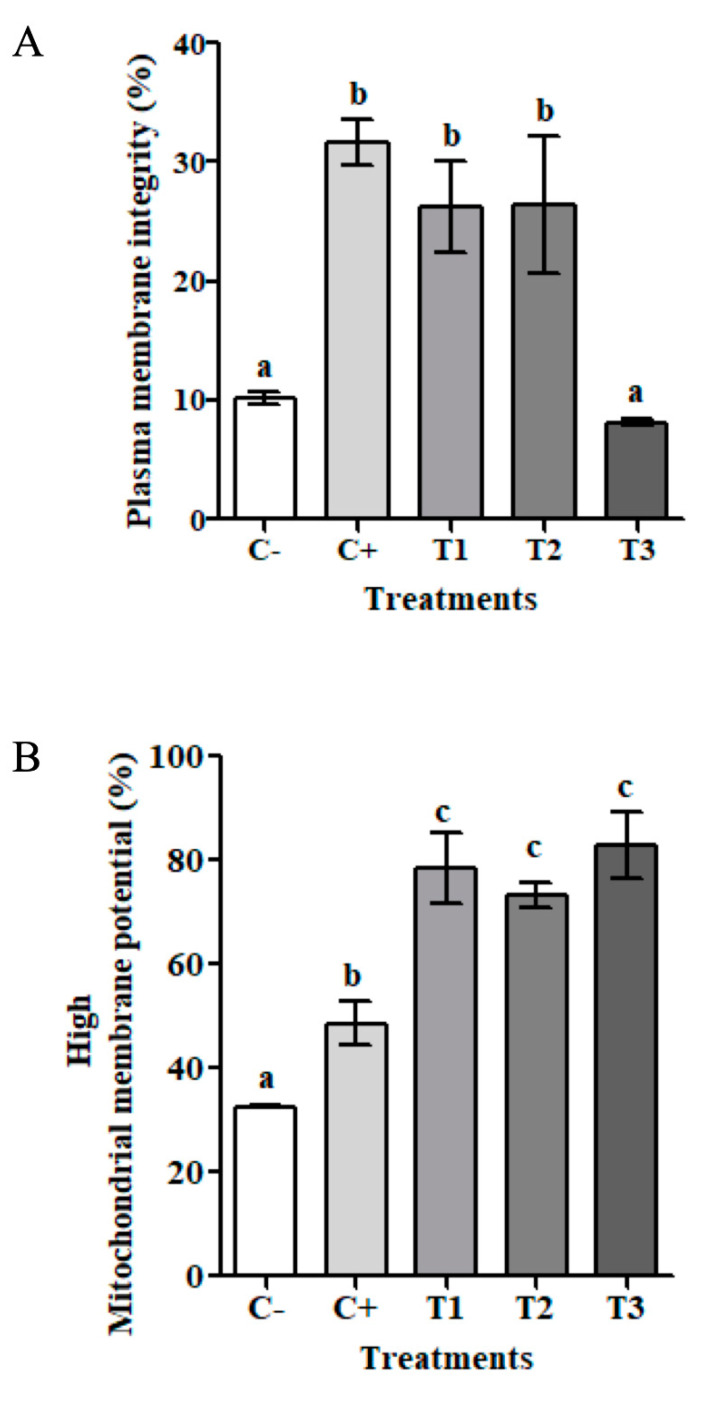
Effect of the cryopreservation on fish sperm quality. Postthawing plasma membrane integrity (PMI) and mitochondrial membrane potential (MMP) analysis. (**A**) Percentage of spermatozoa that maintained PMI postthawing. (**B**) Percentage of spermatozoa with PMI that maintained high MMP postthawing. C−: semen without cryoprotectant; C+: standard freezing medium; T1: 1.3 M Me_2_SO, 0.3 M Glucose, 2% (*w*/*v*) BSA and 0.05 mg/mL apoplastic extract; T2: 1.3 M Me_2_SO, 0.3 M Glucose and 0.05 mg/mL apoplastic extract; T3: 0.05 mg/mL apoplastic extract. One-way ANOVA and Tukey post hoc tests were applied for multiple comparisons. Significance levels are marked with a different letter, where a, b and c are significantly different, *p* < 0.05, *n* = 3.

Atlantic salmon is one of the three most important salmonid species in global aquaculture. We examined the ability of the apoplastic extracts to cryopreserve *S. salar* spermatozoa. Significant differences were observed between spermatozoa cryopreserved in the presence of apoplastic extract supplemented with cryoprotectant (T1 and T2) compared to treatments without cryoprotectant (T3) (Table 1), maintaining a higher percentage of spermatozoa with plasma membrane integrity (PMI) in T1 and T2 (Figure 4A). These treatments were capable of maintaining a similar percentage of spermatozoa with PMI compared to the C+, that is, approximately 30% (Figure 4A). High mitochondrial membrane potential (MMP) is also used as an indicator of successful cell cryopreservation. The percentage of spermatozoa with membrane integrity (~30%) displaying high MMP was significantly higher (80%) in the presence of apoplastic extracts supplemented with cryoprotectants (T1 and T2), and apoplastic extracts (T3) when compared to the C+ (Figure 4B).

Motility assessment was carried out after 30 days of freezing in thawed spermatozoa treated with apoplastic extracts exhibiting antifreeze activity and supplemented with cryoprotectants. Table 2 shows these results. For the C− and T3 (no cryoprotectants) treatments, static spermatozoa and the presence of aggregates were observed. Motility obtained in C+ and T1 was <1%. On the other hand, T2 treatment showed a slight increase in motility, identifying spermatozoa moving at different speeds, with the majority exhibiting slow motility (2.8%). 

## 4. Discussion

IRI has been described as the most sensitive method to determine antifreeze activity since AFPs can inhibit recrystallization of ice at nanomolar concentrations [29]. Nevertheless, this property has not always been detected in all known AFPs. We report here the characterization of the apoplastic protein extracts from *D. antarctica*, observing IRI activity, TH activity, and modification of ice crystal morphology. In addition, we report its preliminary performance as a cryoprotectant.

The lowest apoplastic protein concentration showing IRI activity was 0.05 mg/mL (Figure 1). These results are similar to what has been seen by Doucet et al. (2000) [24], where IRI activity was measured in the aerial parts of *D. antarctica*. Their findings also displayed 0.05 mg/mL as the lowest protein concentration at which IRI activity is detected. The ability to maintain small ice crystal size within a frozen solution is a highly desirable property and has important medical, commercial and industrial applications [5], such as preventing uncontrolled ice crystal growth [45].

Thermal hysteresis activity is one of the most used methods for the identification of antifreeze activity [31,46,47]. It is defined as the depression of the freezing point of a solution relative to its melting point [29]. AFPs produced by freezing-tolerant plants have a low range of TH activity, typically 0.1 to 0.5 °C [31], which is consistent with our results shown in Figure 2 (TH of 0.4 °C at an apoplastic protein concentration of 2.5 mg/mL). The TH activity is directly related to the type and concentration of AFPs in the solution [48], an effect which we observed by decreasing the concentration of extract, resulting in different TH values (0.4–0.05 °C).

Like TH, ice crystal morphology also depends on the concentration and type of AFP [31]. Our results showed a bipyramidal shape at a protein concentration of 2.5 mg/mL (Figure 2). This shape is the typical morphology seen with moderately active AFPs [5]. A study by Drori et al., (2015) [49] showed that moderately active AFPs typically bind to prism faces and/or pyramidal planes of ice crystals and limit their growth without binding to the basal planes, providing the growth of bipyramidal crystals. A lower concentration of apoplastic proteins (0.01 mg/mL) produced ice crystals with flat hexagonal shape (Figure 2). These results are often observed with diluted AFP samples, where proteins bind to the prism faces of ice [50]. 

Freezing media have been described as a group of chemical components capable of maintaining cellular conditions by protecting cells from cryo-damage by decreasing their eutectic point [51]. Given that freezing media can contain both permeable and impermeable cryoprotectants, we evaluated the antifreeze properties of the new AFP-derived extract when combined with conventional cryoprotectants. Our results indicated an interesting effect of IRI activity regarding the ability of apoplastic proteins to inhibit ice crystal growth, which was improved in the presence of the cryoprotectants assessed (Figure 3A). This would indicate what had previously been observed [52] in postthaw sperm results: a possible synergistic effect between cryoprotectants and the apoplastic extract. A similar effect was seen in the TH activity (Figure 3B), which increased by approximately 50% for the combined extract (0.63 °C, Figure 3B), compared to the extract alone (0.4 °C, Figure 2) and even exceeding the value reported for cold-tolerant plants (0.1–0.5 °C) [31]. At the same time, the ice crystal morphology observed in the presence of the combined extract indicates antifreeze activity. This synergy may be explained by the capacity of cryoprotectant to interact with cell membranes through interactions of Me_2_SO with membrane proteins [53], being able to stabilize the protein through interaction with its hydrophobic regions.

PMI is frequently evaluated during routine semen analyses and is a useful predictor of its in vitro fertilizing capacity [54]. In the same way, mitochondria have a physiological requirement for membrane integrity, hence an impermeable inner mitochondrial membrane is required to maintain the ATP synthesis-proton-driven electrochemical gradient and sufficient supply of substrates for various metabolic events occurring inside and outside [44]. For this reason, both PMI and MMP have become key quality indicators of postthawed spermatozoa.

Cryopreservation of *S. salar* spermatozoa was tested with a standard freezing medium and three different treatments with apoplastic extracts supplemented with Me_2_SO, glucose and BSA cryoprotectants, and in the absence of any protectant (Table 1). According to our results, thawed cells in the presence of apoplastic extracts supplemented with cryoprotectants (T1 and T2) showed no significant differences in plasma membrane integrity compared to the C+, maintaining around 30% of cells with their membrane intact (Figure 4A). This is similar to the data shown by Beirão et al. (2012) [55] in *Sparus aurata* spermatozoa and by Zilli et al. (2014) [52] in *Sparidae* spermatozoa where both samples were cryopreserved with Me_2_SO and a purified type I AFP (0.1 μg/mL). Their results displayed between 30 and 40% cell viability and did not observe significant differences between treatments. In salmonid spermatozoa, Me_2_SO, a penetrant cryoprotectant, can maintain plasma membrane integrity, mitochondrial function, and motility patterns when supplemented with antioxidants and plasma membrane stabilizers, such as the nonpermeable cryoprotectant BSA [12]. In agreement with this, our results showed no differences between T1 and T2 treatments, which could explain why our apoplastic extracts could replace BSA. This cryoprotective effect could be attributed to the capacity of adsorption of the AFP present in the apoplastic extract to the surface of ice crystals present in the extracellular space (Figure 4A). Thus, the potential cryoprotectant capacity of these extracts may be improved due to a synergistic effect with the permeable cryoprotectant agent (Me_2_SO) [55]. Accordingly, extracts would have cryoprotectant capacity but may require the presence of a permeating agent to allow for the reduction of water without the deleterious effect caused by changes in the intra- and extracellular space [53,56].

In addition to the protection of PMI, an increase in the percentage of thawed spermatozoa with high MMP (Figure 4B) was observed in T1, T2 and T3 when compared to the C+. This may hint that the apoplastic extract alone may exert a positive effect, as well as a synergic one with the cryoprotectants. Usually, MMP can be used to analyze the mitochondrial status [57], and thus, this parameter may reveal a concomitant effect of the treatments with the motility function of *S. salar* spermatozoa. Moreover, our results indicate that the apoplastic extract alone is able to improve the high MMP percentage above C+, showing that the extract itself contributes to maintaining high MMP in *S. salar* spermatozoa. 

Total motility measured revealed levels below 1% in most treatments, which is an unexpected outcome considering the percentage of spermatozoa with high MMP. Nonetheless, results by Zilli et al., (2014) [52] have shown that the reduction in the expression of glyceraldehyde-3-phosphate dehydrogenase and malate dehydrogenase (enzymes whose catalytic process generates NADH cofactor necessary for ATP production) in spermatozoa cryopreserved in the presence of Me_2_SO may contribute to the observed reduction in sperm motility after the freeze–thaw procedure. Despite the fact that these results may seem low, short- and long-term freezing protocols severely impede motility in *S. salar*. A study by Erraud et al., (2021) displays 5–10% motile sperm after just one day frozen [58]. In addition, Dziewulska et al., (2011) assessed eight different cryopreservation protocols, and their results reached values close to ours in most of these treatments [59].

In this work, we assessed the effect of *D. antarctica* apoplastic extract on fish sperm cryopreservation and offered evidence to demonstrate its potential influence on cryobiology. The work of Le François et al., (2008) [60] revealed that the natural expression of AFPs in the seminal fluid of Atlantic wolffish (*Anarhichas lupus*) provides an increased fish sperm resistance to cryopreservation. Their research forms the basis for the assertion that these proteins may potentially serve as a method for cryopreserving sperm from various fish species. In addition, according to the work of Prathalingam et al., (2006) [33] in bull sperm preservation, one potential way in which AFPs may function is by accumulating and interacting with the surface of ice crystals and plasma membranes of sperm and in due term affecting crystals morphology parameters. The results of our study with the apoplastic extract of *D. antarctica* are in accordance with their results, supporting the presence of AFPs in the extract. In addition, crystal morphology, TH, IRI assays provided support for the use of this extract as part of freezing media for cryopreservation. Thus, the effect of *D. antarctica* apoplastic extract on fish sperm cryopreservation was assessed. These results displayed that adding apoplastic extracts to the freezing medium allows maintaining cell integrity of thawed spermatozoa. In addition, these demonstrated that during the freezing and thawing procedure, apoplastic protein extracts could prevent ice crystal growth in extracellular space, preventing their growth and concomitant damage to the spermatozoa membrane. However, parameters of motility postthaw have been a challenge, and there is a need for improvement of the storage time and sample number. Considering the indicators mentioned (IMM, PMM, and motility), we have so far managed to maintain conditions equivalent to standard cryopreservation media. Finally, our results suggest that the AFP present in the extract may be able to replace BSA in traditional freezing media and may have a synergetic effect when used together with a permeable cryoprotectant. These findings represent the first report that suggests that AFPs derived from apoplastic extracts of *Deschampsia antarctica* have the potential to serve as cryoprotectants and could allow the development of novel freezing media. We believe that a strategy using *D. antarctica* AFPs as a replacement for traditional external cryoprotectants may be developed. However, our results are preliminary, and further research is required to sustain this hint, i.e., allowing to provide higher PMI and MMP postthaw.

## Figures and Tables

**Table 1:** Description of controls and treatments used for the cryopreservation process.

Controls and Treatments	Description
**C−**	Semen diluted in Cortland^®^ medium in the ratio 1:3 (semen: Cortland^®^) without cryoprotectants.
**C+**	Semen diluted in Cortland^®^ medium supplemented with 1.3 M Me_2_SO; 0.3 M glucose and 2% (*w*/*v*) BSA in the ratio 1:3 (semen: freezing medium).
**T1**	Semen diluted in Cortland^®^ medium supplemented with 1.3 M Me_2_SO; 0.3 M glucose, 2% (*w*/*v*) BSA and 0.05 mg/mL apoplastic extract in the ratio 1:3 (semen: freezing medium).
**T2**	Semen diluted in Cortland^®^ medium supplemented with 1.3 M Me_2_SO; 0.3 M glucose and 0.05 mg/mL of apoplastic extract in the ratio 1:3 (semen: freezing medium).
**T3**	Semen diluted in Cortland^®^ medium supplemented with 0.05 mg/mL apoplastic extract in the ratio 1:3 (semen: freezing medium).

**Table 2:** Sperm motility of *S. salar* postthaw in the presence of apoplastic extracts from *D. antarctica* supplemented with cryoprotectants.

Controls and Treatments	Total Motility (%)
C−	100 (E)
C+	0–1 (L)
T1	0–1 (L)
T2	2.8 (L); 0.80 (M); 0.22 (R)
T3	100 (E)

C−: Semen without cryoprotectant. C+: Standard freezing medium. T1: DMSO 1.3 M, glucose 0.3 M, 2% *w*/*v* BSA, and 0.05 mg/mL apoplastic extract. T2: DMSO 1.3 M, glucose 0.3 M, and 0.05 mg/mL apoplastic extract. T3: 0.05 mg/mL apoplastic extract. (E): Static spermatozoa; (L): Spermatozoa with slow movement; (M): Spermatozoa with medium movement; (R): Spermatozoa with rapid movement.

## Data Availability

The data that support the findings of this study are available from the corresponding author, [J.G.F.], upon reasonable request.
